# Time-point-based analysis of gold nanoparticles in MCF-7 cells following ultrasound irradiation: quantitative and label-free intracellular characterization

**DOI:** 10.1039/d5na00620a

**Published:** 2026-04-08

**Authors:** Jiwon Kim, Chaewon Bae, Rodrigo Hernández Millares, Taeyun Kim, Yejin Lee, Kangwon Lee, Sung-Joon Ye

**Affiliations:** a Department of Applied Bioengineering, Graduate School of Convergence Science and Technology, Seoul National University Seoul 08826 Republic of Korea sye@snu.ac.kr; b Program in Nanoscience and Technology, Graduate School of Convergence Science and Technology, Seoul National University Seoul 08826 Republic of Korea; c Advanced Institute of Convergence Technology, Seoul National University Suwon 16229 Republic of Korea; d Biomedical Research Institute, Seoul National University Hospital Seoul 03080 Republic of Korea

## Abstract

Ultrasound-mediated nanoparticle delivery has garnered increasing attention in recent years; however, the time-lapse intracellular distribution of gold nanoparticles (GNPs) following ultrasound exposure has not been adequately explored yet, particularly with respect to quantitative uptake and optimized ultrasound parameters. Here, we provide analysis addressing these gaps by employing quantitative and label-free intracellular tracking to elucidate the interactions between ultrasound and GNPs in MCF-7 breast cancer cells. MCF-7 cells treated with GNPs were exposed to 40 kHz ultrasound for 5, 10, and 20 min. Following irradiation, the cells were incubated for 0, 3, or 24 h to assess post-ultrasound intracellular distribution. Confocal imaging was performed to visualize label-free intracellular GNP clusters, enabling spatial analysis of nanoparticle dispersion. Ultrasound irradiation reduced the maximum cluster size of intracellular GNPs with increasing exposure time, reaching up to a 22% decrease compared to the control group. Optical diffraction tomography further revealed sonoporation upon ultrasound exposure, indicating ultrasound-induced membrane permeabilization. GNP concentrations measured by inductively coupled plasma atomic emission spectroscopy demonstrated that GNP uptake increased immediately post-irradiation, showing a 3.5-fold rise in the 20 min group. However, this enhancement diminished over time, with intracellular GNP levels across all groups converging around 10 h post-treatment. Nonetheless, ultrasound promoted a more uniform cytoplasmic distribution of GNPs. These findings highlight ultrasound as a rapid yet transient enhancer of intracellular GNP delivery and dispersion. Our label-free and quantitative approach enabled the spatiotemporal assessment of GNP dynamics, revealing time-dependent uptake and sonoporation.

## Abstract 2

The intracellular diffusion and accumulation of gold nanoparticles are characterized as functions of ultrasound irradiation and recovery time. GA made with use Biorender.

## Graphical abstract

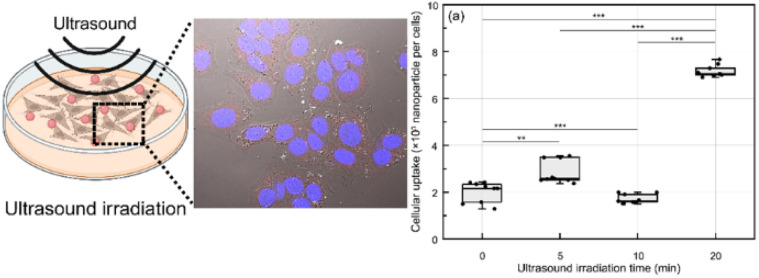

## Introduction

Targeted drug delivery to specific cells or tissues can enhance treatment efficacy while reducing systemic side effects. Unlike conventional methods that disseminate drugs throughout the entire body, targeted delivery leverages specific mechanisms to concentrate therapeutic agents precisely at the intended site.^1–3^ Nonetheless, targeted drug delivery faces several challenges. A key limitation is the restricted accumulation of drugs at the target site, often due to biological barriers, such as the tumour microenvironment and the blood–brain barrier, which impede drug penetration and distribution.^4–9^ Additionally, limited specificity can further compromise therapeutic efficacy.^10,11^ Furthermore, the tendency of nanoparticles to aggregate during systemic circulation or within cells may limit their therapeutic efficacy, hindering uniform drug distribution.^12^ Drugs, especially used in cancer treatment, not only exhibit limited accumulation at tumour sites but also face functional limitations that can reduce treatment efficacy.^13^ To improve therapeutic efficacy, there is a growing demand for multifunctional agents, which has led to the use of nanoparticles such as gold nanoparticles (GNPs). GNPs are particularly well-suited for targeted delivery due to their biocompatibility, ease of surface functionalization, and capacity to be conjugated with a wide range of therapeutic and diagnostic molecules.^14–16^ However, research is still underway to determine the most effective nanoparticles and functional markers for optimal outcomes, with experimental and clinical studies on nanoparticle behaviour remaining unstandardized and in need of further optimization.

To address the limited accumulation and distribution of drugs at the target site, ultrasound-mediated drug delivery has emerged as a promising alternative. Through sonoporation, which creates temporary pores in the cell membrane, ultrasound can increase drug permeability and retention within tumours, supporting a more effective, localized therapeutic approach.^17^ Although this effect is short-lived (*e.g.*, a few min), it can enhance the delivery of nanoparticles and therapeutic agents into cells.^18,19^ These temporary pores allow nanoparticles as drug carriers to enter the cells, improving drug delivery efficiency.^20^ Moreover, ultrasound prevents intracellular nanoparticle aggregation and enhances cytoplasmic dispersion through cavitation, which creates microbubbles that induce localized mechanical forces.^21^ These forces help break up nanoparticle clusters, allowing for more uniform distribution within the cell and potentially improving the effectiveness of drug delivery.^10,22^ However, several challenges persist in optimizing ultrasound parameters such as frequency, intensity, and irradiation duration to achieve effective delivery without inducing off-target site damage.^23–27^

**Fig. 1:**
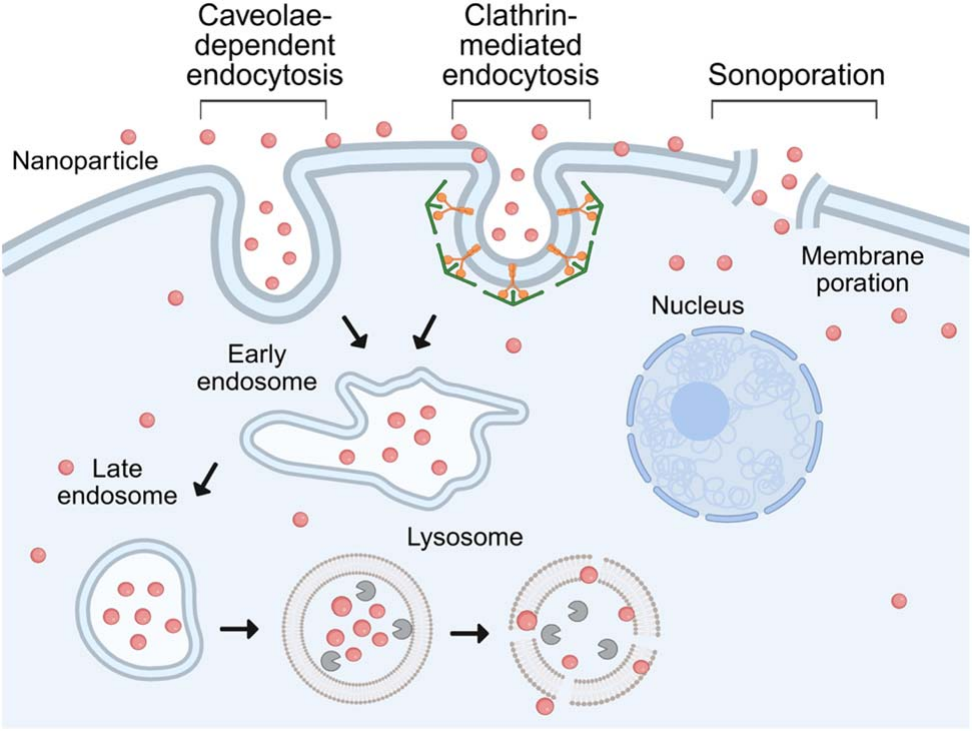
Schematic illustration of the endocytosis pathway induced by ultrasound irradiation on cells, including caveolae-dependent endocytosis, clathrin-mediated endocytosis, and sonoporation for enhanced endocytosis and drug delivery. Created with http://BioRender.com.

Efforts to optimize ultrasound parameters and achieve uniform drug distribution in ultrasound-mediated drug delivery have been widely documented.^28–30^ Studies have investigated a range of ultrasound frequencies and intensities, aiming to enhance drug penetration while minimizing adverse effects on off-target sites.^21,31^ Early ultrasound research primarily focused on frequencies in the MHz range.^32–35^ Ultrasound irradiation at higher frequencies has been shown to enhance drug uptake by increasing cell membrane permeability. Nonetheless, it can also induce microbubble formation, which generates reactive oxygen species, potentially causing oxidative stress. Combined with the risk of mechanical damage to adjacent healthy tissues, this raises concerns about increased treatment-related toxicity.^36^ Therefore, more recently, studies have shifted towards exploring low-frequency ultrasound in the kHz range, as it has been found to be more effective for enhancing intracellular uptake of therapeutic agents with reduced adverse effects.^37,38^ In the realm of medical ultrasound applications, precise control over ultrasound frequencies is paramount. The 40 kHz ultrasound frequency is advantageous, offering cost-effectiveness and ease of manipulation relative to other frequencies. These attributes contribute to the widespread adoption of 40 kHz ultrasound in many standard low-frequency ultrasound transducers. The application of 40 kHz ultrasound induces cavitation and sonoporation, facilitating the formation of pores in cellular membranes without causing tissue damage.^39^ Previous studies have shown a significant enhancement in the efficacy of nanoparticle delivery using 40 kHz ultrasound.^40,41^ Upon ultrasound irradiation, multiple cellular uptake pathways are activated, including caveolae-dependent endocytosis, clathrin-mediated endocytosis, and sonoporation (Fig. 1). The internalized nanoparticles are typically transported through the endosomal pathway and ultimately accumulate in lysosomes. Ultrasound can increase the permeability of both the plasma and lysosomal membranes, which facilitates the disaggregation of nanoparticle clusters.^42^ Thereby, the intracellular dispersion of nanoparticles is enhanced and the size of the clusters is reduced. The reduction in nanoparticle cluster size increases the overall surface area, potentially enhancing intracellular mobility and improving the efficiency of nanoparticle-mediated drug delivery. Large nanoparticle aggregates may become biologically inactive within cells, whereas the disaggregation and dispersion of these clusters may restore or even enhance their therapeutic function. Importantly, clustered nanoparticles are more prone to lysosomal sequestration, which can contribute to cytotoxicity and long-term toxicity.^43,44^ Ultrasound facilitates the breakdown of these aggregates, promoting rapid decomposition and release of individual nanoparticles, thereby potentially mitigating lysosome-associated toxicity. The ability of ultrasound to disaggregate intracellular nanoparticle clusters represents a key therapeutic advantage, enabling improved distribution, reduced toxicity, and the potential for enhanced functional performance of nanomaterials in biomedical applications.

The accurate quantification and analysis of drug uptake and retention in cells remains a major hurdle for ultrasound-mediated cancer treatment.^21^ Tracking intracellular nanoparticles using fluorescence-based techniques has become a common strategy to resolve these challenges.^15,20,30,45^ Nonetheless, the use of fluorescent agents may alter the intrinsic properties of the nanoparticles and is prone to photobleaching, posing limitations for imaging.^46,47^ Unlike fluorescence-based techniques, label-free reflectance confocal microscopy enables direct observation of metallic nanoparticles such as GNPs without the need for chemical tagging.^46,48^ This non-invasive approach allows an accurate assessment of nanoparticle distribution within cells, preserving the physiological context and minimizing artifacts.^49^ Although ultrasound irradiation has demonstrated enhancement in cellular uptake of nanoparticles, the long-term persistence of this effect remains unexplored.^23^ Label-free imaging techniques offer a promising approach to more accurately assess nanoparticle uptake while minimizing potential interference with cellular function and nanoparticle behaviour.

In this study, we investigated the spatiotemporal behaviour of GNPs in MCF-7 breast cancer cells following low-frequency ultrasound irradiation. The intracellular accumulation of GNPs was quantitatively assessed by inductively coupled plasma atomic emission spectroscopy (ICP-AES) at multiple time points (0, 3, and 24 h) to characterize uptake behaviour over time. A label-free imaging approach was employed to visualize the intracellular distribution and aggregation state of internalized GNPs. In addition, morphological changes in cell membrane integrity were evaluated using optical diffraction tomography (ODT) to determine the extent of ultrasound-induced permeabilization. Through the integration of quantitative and morphological analyses, our findings demonstrated that ultrasound facilitates transient membrane disruption and promotes the dispersion of GNPs, resulting in a more uniform and temporally regulated intracellular distribution.

## Results and discussion

### GNP characterization

**Fig. 2:**
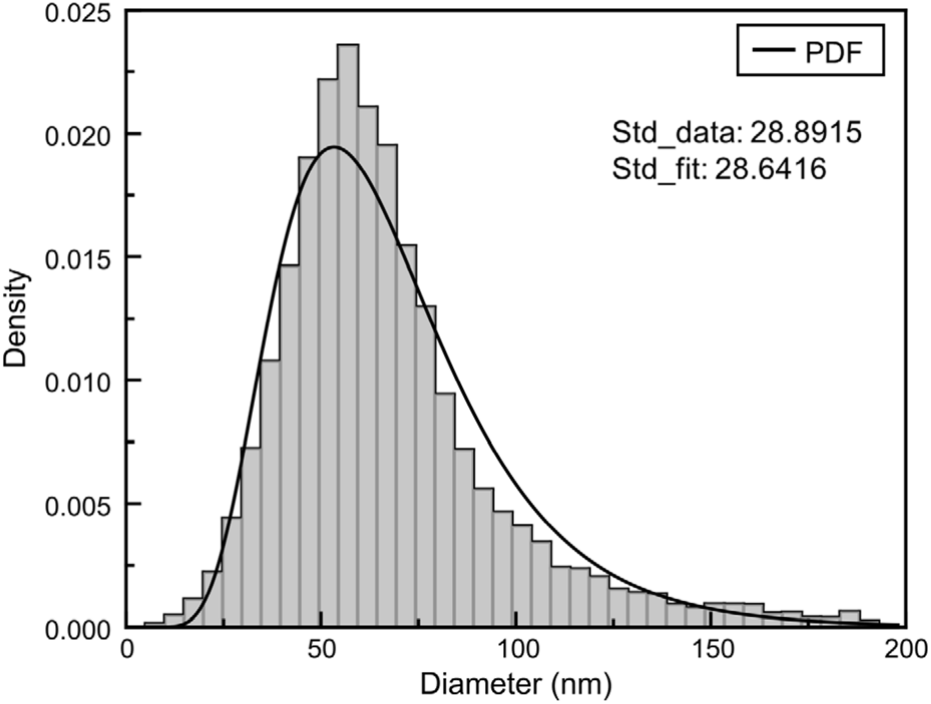
Size distribution of GNPs as measured by NTA. The distribution spans from 20 nm to 200 nm in diameter, with the highest particle concentration occurring at 57 nm. The total particle concentration was measured as 9.28 × 10^8^ particles per mL. The solid line represents the calculated probability density function (PDF) based on the log-normal fit of the measured data.

Fig. 2 shows the size distribution and concentration of GNPs measured using Nanoparticle Tracking Analysis (NTA). For size analysis, the GNP solution was diluted to a concentration of 1 × 10^9^ particles per mL using deionized water. The particles exhibited a broad distribution ranging from approximately 20 nm to 200 nm in diameter. A prominent peak in particle concentration was observed at 57 nm. This peak diameter reflects the most abundant population within the sample. The total particle concentration was determined to be 9.28 × 10^8^ particles per mL. The measured modal diameter of the GNPs closely aligns with the manufacturer's reported value of 55 nm, as provided by Sigma-Aldrich.^50^

### Cell counting kit-8 (CCK-8) assay

**Fig. 3:**
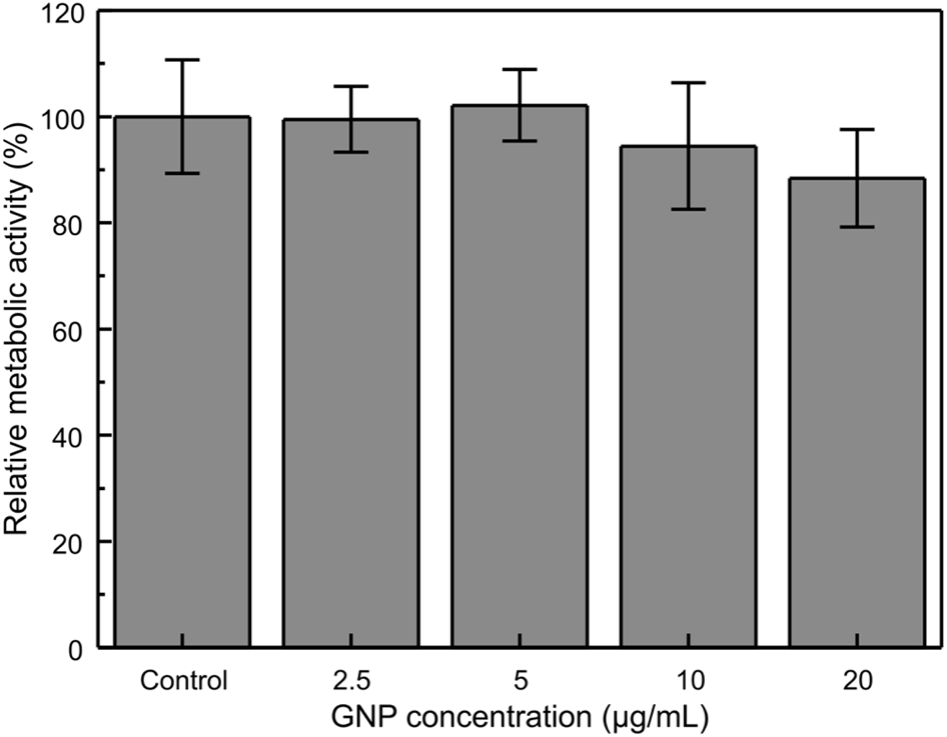
Relative metabolic activity of MCF-7 cells at different GNP concentrations. MCF-7 cells were incubated with GNPs for 24 h (*n* = 3).

To assess the cytotoxicity of GNPs to MCF-7 cells, various concentrations of GNPs were administered to the cell media. The cell viability after GNP administration is a crucial factor in determining the maximum nanoparticle concentration for enhanced drug delivery. Furthermore, to obtain reliable data on intracellular GNP levels, maintaining a high cell number is critical for accurate quantification. Groups were compared using one-way ANOVA and subsequent Tukey's HSD *post hoc* analysis. This multiple comparison test revealed no significant difference in relative metabolic activity across all treatment groups (Fig. 3). To enhance cellular uptake, increasing the nanoparticle concentration in the media is typically advantageous. For subsequent experiments, a maximum concentration of 20 µg mL^−1^ was selected to improve drug delivery efficiency at the target site while minimizing potential cytotoxic effects.

### Intracellular distribution of GNPs visualized by label-free imaging

**Fig. 4:**
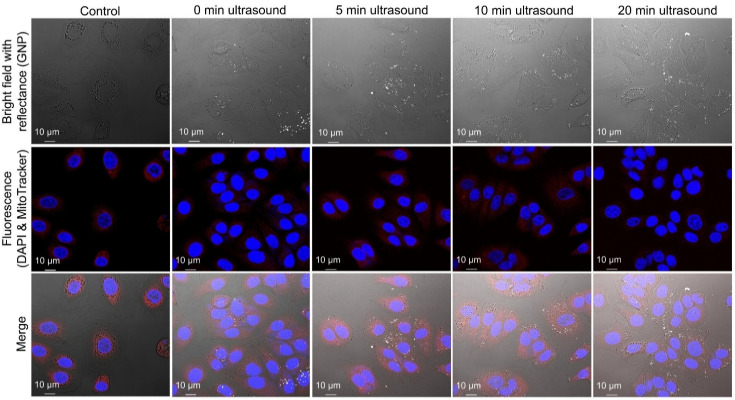
Label-free confocal reflectance images of intracellular GNPs in MCF-7 cells under ultrasound irradiation conditions. GNPs are absent in the control images, while distinct nanoparticles are observed in the experimental groups. GNPs appear as bright reflectance signals dispersed within the cytoplasm.

Label-free confocal reflectance images present brightfield, reflectance, and fluorescence images of MCF-7 cells following ultrasound exposure (Fig. 4). In the absence of ultrasound, GNPs tended to form relatively large clusters. These images are two-dimensional (2D) projections, and although nanoparticles may appear to be located within the nucleus, this is an artifact of imaging in a single focal plane. Our setup achieved a resolution of 200 nm by minimizing the pinhole size and utilizing a 60× oil immersion objective. Although z-stack imaging confirmed the absence of GNPs within the cell nucleus, subsequent image post-processing made it appear as though GNPs were distributed inside the nucleus. This phenomenon is an inherent limitation of imaging GNPs using 2D through mode, as the signal from GNPs located near the nuclear membrane can blur axially, causing them to appear superimposed over the nucleus in a single 2D image. This limitation is present when imaging GNPs in reflectance mode in 2D. By employing label-free imaging, the physiological context was preserved and potential artifacts associated with chemical labeling were minimized. Since chemical tags can alter the physicochemical properties of nanoparticles, their absence enables a more accurate evaluation of the interaction between ultrasound exposure and nanoparticle distribution.

**Fig. 5:**
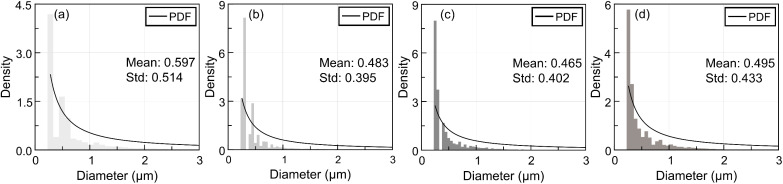
Intracellular GNP cluster size histogram after ultrasound irradiation. (a) 0 min ultrasound irradiation, (b) 5 min ultrasound irradiation, (c) 10 min ultrasound irradiation and (d) 20 min ultrasound irradiation.

Following ultrasound irradiation, a marked change in the distribution and clustering behaviour of GNPs was observed. Across all ultrasound exposure times (5, 10, and 20 min), GNP cluster sizes were observed to be more decreased throughout the cytoplasm compared to the non-irradiated control group (Fig. 5). The average size of the GNP clusters was reduced immediately after ultrasound treatment, suggesting that ultrasound can enhance intracellular dispersion of GNPs. Quantitative analysis revealed that the mean cluster size was calculated from the cluster sizes of ten representative images in each group. The mean cluster size of the 0 min group was 0.597 ± 0.514 µm. In contrast, GNP cluster sizes were reduced in ultrasound-irradiated cells: 0.483 ± 0.395 µm for the 5 min group, 0.465 ± 0.402 µm for the 10 min group, and 0.495 ± 0.433 µm for the 20 min group. These correspond to reductions of 19.1%, 22.11%, and 17.09%, demonstrating enhancement in nanoparticle dispersion with ultrasound irradiation. To evaluate the dependence between the average particle size and the ultrasound exposure time for each group, several statistical models were employed for analysis. Analysis of the linear correlation using the entire set of individual measurement data yielded a Pearson correlation coefficient (*r*) of −0.0278 and a *p*-value of 9.9478 × 10^−19^. Since the *p*-value was significantly lower than the significance level, we confirmed a statistically significant linear dependence between the ultrasound treatment time and the size of the individual particles. However, the correlation strength, as indicated by the low *r* value, was weak, suggesting that the model was insufficient to fully explain the underlying physical phenomena. This limitation arose because the high variance within the groups was included in the overall model. Therefore, we additionally performed a quadratic regression analysis based on the mean values, which better reflects the physical phenomena of particle dispersion and aggregation. The coefficient of determination (*R*^2^) for the linear model was 0.3392, while the *R*^2^ for the quadratic model was 0.9579, with a *p*-value of 0.1624. Although the *p*-value of the quadratic model failed to reach the significance level, primarily due to the limitation of a small sample size (*n* = 4), the high *R*^2^ value strongly supports that the model effectively explains the variability in particle size as a function of increasing ultrasound exposure time.

Moreover, a progressive decrease in the maximum cluster size was observed with increasing ultrasound exposure time, suggesting a time-dependent enhancement in nanoparticle dispersion. These findings indicate that prolonged ultrasound exposure results in a more fragmented and widespread intracellular distribution in the perinuclear region, reflecting enhanced intracellular dispersion and reduced aggregation. Ultrasound-induced dispersion of GNPs likely contributed to improved therapeutic efficiency by promoting a more uniform intracellular distribution as reported earlier.^20,51^ This uniform distribution may enhance therapeutic efficiency, broaden the area of drug action within the cytoplasm, and reduce localized nanoparticle accumulation, thereby minimizing potential cytotoxicity. However, the label-free confocal imaging provided only 2D projections, which may not accurately reflect the precise 3D spatial distribution of intracellular GNPs.

To better understand the underlying mechanisms, known uptake pathways were considered. Upon exposure to ultrasound, this uptake pathway is further enhanced, particularly *via* caveolae-dependent endocytosis, clathrin-mediated endocytosis,^42^ and sonoporation as previously reported. Unmodified GNPs tend to aggregate and are typically trapped within endosomes following endocytosis. Early endosomes are generally observed near the plasma membrane shortly after internalization, whereas late endosomes are located closer to the nucleus^52^ (Fig. 1). Over time, GNPs are trafficked to lysosomes, which are predominantly distributed in the perinuclear region but can also be found throughout the cytoplasm. This localization is consistent with prior studies showing lysosomal transport along microtubules toward the perinuclear area.^53^

In addition to influencing uptake pathways, ultrasound may transiently increase the permeability of endosomal and lysosomal membranes, thereby modifying internal pH and ionic environments.^54^ Such changes could restore electrostatic repulsion between GNPs and facilitate the disaggregation of nanoparticle clusters. This ultrasound-induced re-dispersion mechanism provides a non-invasive means of improving the intracellular mobility of nanomaterials and mitigating the long-term toxicity associated with lysosomal sequestration. Importantly, the application of label-free imaging in this context represents a novel approach that enables artifact-free visualization of nanoparticle behaviour post-ultrasound treatment. This approach offers new insights into intracellular dispersion dynamics and enhances the translational relevance of these findings. Ultrasound irradiation resulted in a redistribution of intracellular GNPs toward a more dispersed pattern. While this redistribution may influence intracellular delivery dynamics, our current data are not enough to provide direct evidence that a more uniform distribution causally enhances uptake; mechanistic studies including endosomal assays and live-cell tracking are underway.

### GNP quantification after ultrasound irradiation

**Fig. 6:**
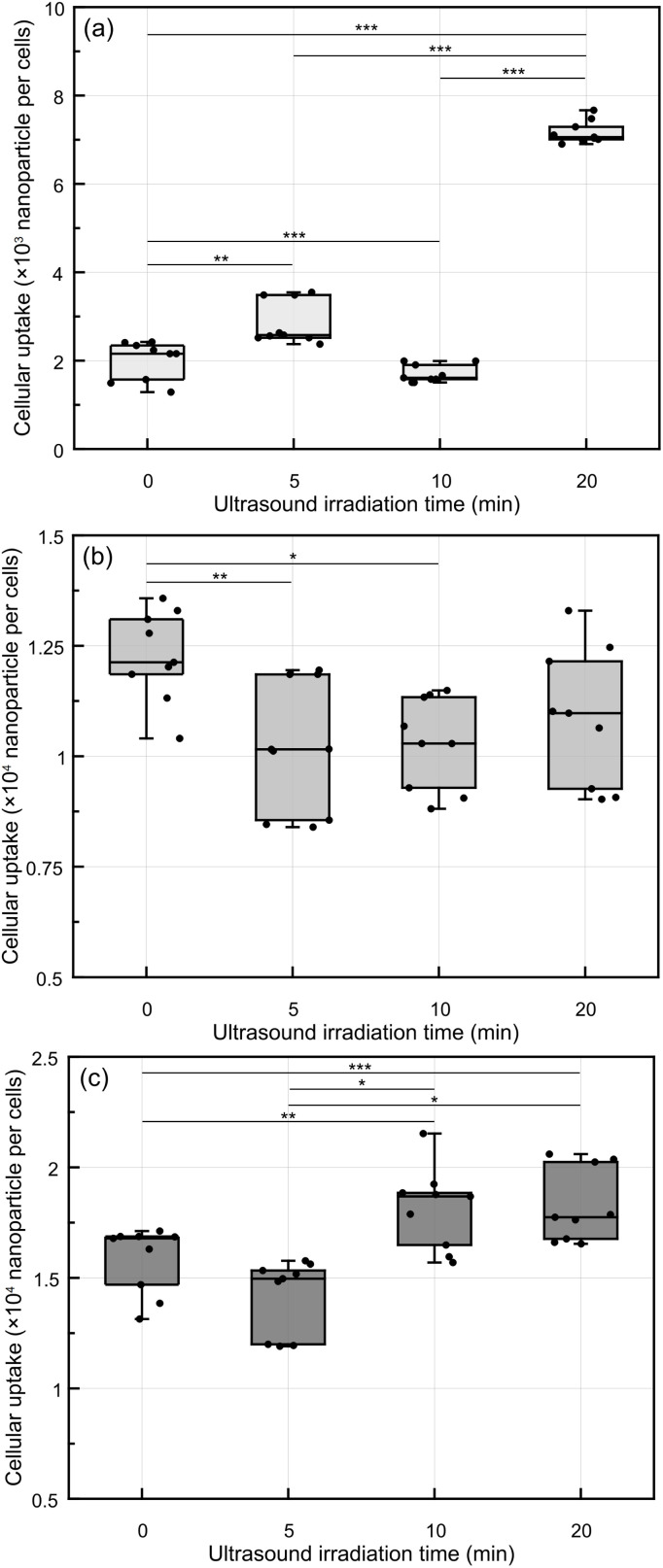
Intracellular uptake of GNP after ultrasound irradiation by ICP-AES. (a) Immediately after ultrasound irradiation, (b) 3 h after ultrasound irradiation, and (c) 24 h after ultrasound irradiation (*: *p* < 0.05, **: *p* < 0.01, ***: *p* < 0.001, *n* = 3).

Intracellular gold element measurement was performed using ICP-AES. Fig. 6 shows the concentration of intracellular GNPs following ultrasound irradiation. Immediately after ultrasound irradiation, there was a marked increase in intracellular GNP uptake corresponding to the time of ultrasound irradiation (Fig. 6a). Notably, the cells exposed to ultrasound for 20 min exhibited a 3.5-fold increase in GNP uptake compared to the non-irradiated cell group. Additionally, GNP internalization in the cells subjected to 5 min of ultrasound was slightly higher than in the non-irradiated cells.

Following this initial response, a 3 h post-irradiation incubation period revealed a general increase in intracellular GNP accumulation under all experimental conditions (Fig. 6b). In fact, GNP uptake in the ultrasound-irradiated cells was slightly lower than in the non-irradiated cells. The quantity of GNPs after 3 h was similar between the ultrasound-irradiated groups. After 24 h post-ultrasound irradiation, there was an increase in GNP uptake in the ultrasound-irradiated cells (Fig. 6c). Ultrasound irradiation for 10 and 20 min continued to influence cellular uptake even after 24 h, resulting in approximately a 10% increase in GNP uptake in the ultrasound-irradiated groups compared to the non-irradiated cells. Table 1 provides a comprehensive overview of the total internalized GNP levels across the three post-ultrasound irradiation intervals for each ultrasound irradiation condition.

**Table 1:** ICP-AES analysis of intracellular GNPs in MCF-7 cells after ultrasound irradiation; post-ultrasound irradiation time (a), ultrasound irradiation time (b), mean gold element concentration of cell groups (*C*_m_), and GNP concentration calculated using the equation (*C*_eq_) (results are represented with mean ± standard deviation (SD))

(b)	*C*_m_ mg L^−1^ (mean ± SD)			*C*_eq_ 10^4^ particle (mean ± SD)		
	(a)					
	0 h	3 h	24 h	0 h	3 h	24 h
0 min	0.25 ± 0.05	1.55 ± 0.13	2.00 ± 0.19	0.20 ± 0.04	1.22 ± 0.10	1.58 ± 0.15
5 min	0.36 ± 0.06	1.29 ± 0.18	1.79 ± 0.21	0.28 ± 0.05	1.02 ± 0.15	1.42 ± 0.17
10 min	0.23 ± 0.02	1.30 ± 0.13	2.29 ± 0.23	0.17 ± 0.02	1.03 ± 0.13	1.81 ± 0.19
20 min	0.91 ± 0.03	1.38 ± 0.19	2.31 ± 0.21	0.72 ± 0.02	1.08 ± 0.15	1.83 ± 0.17

To better understand the dynamics of GNP uptake, a time-dependent analysis was performed. Notably, immediately after ultrasound irradiation, there was a 3.5-fold increase in GNP uptake with 20 min of ultrasound irradiation compared to the control group, though the relationship between ultrasound irradiation duration and cellular uptake was not strictly linear. As shown in previous studies, sonoporation temporarily opens pores in the cell membrane, which persist for a few minutes.^55,56^ This transient effect likely accounts for the pronounced increase in GNP uptake immediately following ultrasound irradiation, with nanoparticle internalization continuing through both ultrasound-enhanced and natural uptake mechanisms even after the initial effect has subsided.

**Fig. 7:**
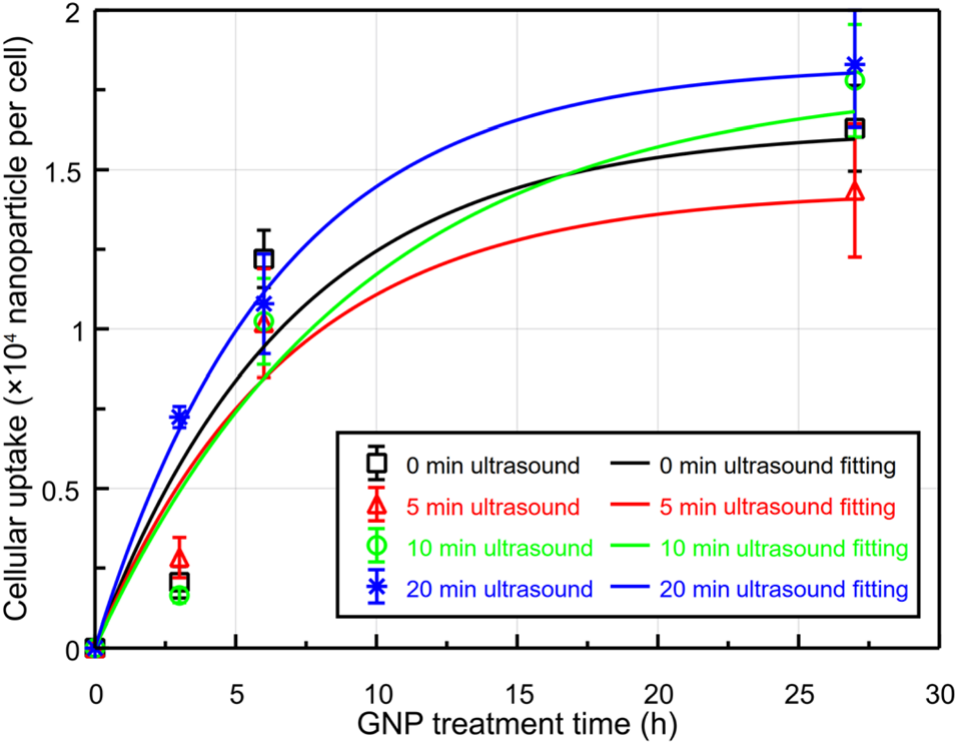
Time-dependent cellular GNP uptake. Due to the 3 h GNP treatment time prior to ultrasound irradiation, the total GNP treatment time was 3, 6, and 27 h. Ultrasound was irradiated on MCF-7 cells for 0, 5, 10, and 20 min following a 3 h GNP treatment time. The first-order absorption model is fitted to the nanoparticle-response data.

However, after 3 h post-ultrasound irradiation, the influence of ultrasound on GNP uptake decreased, and the effect of natural cellular uptake became more dominant. The results of ICP-AES were fitted using a first-order absorption model, with the assumption that no intracellular nanoparticles were present at 0 h (Fig. 7). The results indicated that 20 min of ultrasound exposure markedly increased the initial uptake of nanoparticles compared to other treatment groups; however, the uptake pattern subsequently converged with the other groups over time. To statistically analyze this behavior and ensure both model parsimony and the significance of the absorption rate constant (*k*), the uptake measured 24 h post-treatment was defined as the maximum uptake capacity. With three degrees of freedom (DF = 3), the sole variable *k* yielded a *p*-value <0.05 (Table 2), thereby providing robust statistical support for our claims. Furthermore, the fitting results of the group with the longest ultrasound exposure duration revealed that ultrasound not only significantly accelerates the intracellular uptake rate but also minimizes the random variability within the cellular system. The fitting analysis demonstrated high statistical significance, characterized by exceptionally low *p*-values and high *R*^2^ values, further strengthening the reliability of these findings.

**Table 2:** Statistical parameters for the first-order absorption model for different ultrasound irradiation times. The *p*-values and *R*^2^ values represent the goodness-of-fit for each group

	Coefficient of determination (*R*^2^)	*p*-Value for the model	*p*-Value for *k*
0 min	0.8855	0.015	0.0646
5 min	0.9350	0.0062	0.0279
10 min	0.9275	0.0073	0.0386
20 min	0.9980	3.14 × 10^−5^	0.000131

The trend in intracellular uptake of GNPs varies with time following ultrasound irradiation, with a rapid increase in nanoparticle internalization observed only within a few hours post-irradiation. In the control group, intracellular GNP levels remain relatively stable up to 24 h.^57,58^ Conversely, with 20 min of ultrasound irradiation, there is a substantial and rapid uptake of GNPs immediately post-ultrasound irradiation. With a significant intracellular uptake of GNPs induced by ultrasound irradiation, the rate of natural uptake is notably lower compared to that of other cell groups. ICP-AES analysis further confirmed that the internalization process followed similar patterns across the different groups, with ultrasound-enhanced GNP uptake immediately following irradiation. This pattern converged with that of non-irradiated cells as time progressed. These findings indicate that although natural endocytosis becomes the dominant mechanism over time, the initial ultrasound-induced uptake and dispersion of nanoparticles within the cytoplasm may still contribute positively to therapeutic efficacy by promoting a more uniform intracellular distribution of therapeutic agents.

Ultrasound can enhance nanoparticle internalization in cells,^59–61^ but the cellular uptake of other cellular components must be taken into account when optimizing the effectiveness of ultrasound treatments in cancer cells. In short-term uptake studies, a significant increase in GNP internalization was observed with prolonged ultrasound exposure (20 min). Despite the increasing predominance of natural uptake over time, this ultrasound-induced disaggregation suggests that ultrasound can modulate GNP size in a non-invasive manner. For long-term intracellular uptake, GNP concentrations appear to be driven predominantly by spontaneous endocytic processes, with ultrasound irradiation exerting a relatively minor impact on the final concentration.

### Confirmation of sonoporation *via* ODT

Sonoporation induces pore formation in the cell membrane, leading to an increase in the cell surface area due to the pores formed in the membrane. The occurrence of sonoporation was assessed by comparing cell surface areas before and after ultrasound irradiation. Given the variability in cell size detected through ODT, the membrane area and total cell volume were utilized to calculate the area-to-volume ratio as a measure for assessing sonoporation. The area-to-volume ratio was acquired based on the ODT images. This study presents a novel application of ODT to measure ultrasound-induced sonoporation in cells. Specifically, this approach establishes a mechanistic link by demonstrating how morphological alterations evolve as a function of ultrasound irradiation and post-irradiation time, which directly correlate with the sonoporation process. The surface area-to-volume ratio serves as a robust supplementary indicator to elucidate the mechanical changes resulting from the synergistic interaction between nanoparticle treatment and ultrasound irradiation. The ODT-based analysis enables the rapid acquisition of large-scale cellular data, providing a comprehensive understanding of population-level trends and mechanical shifts.

**Fig. 8:**
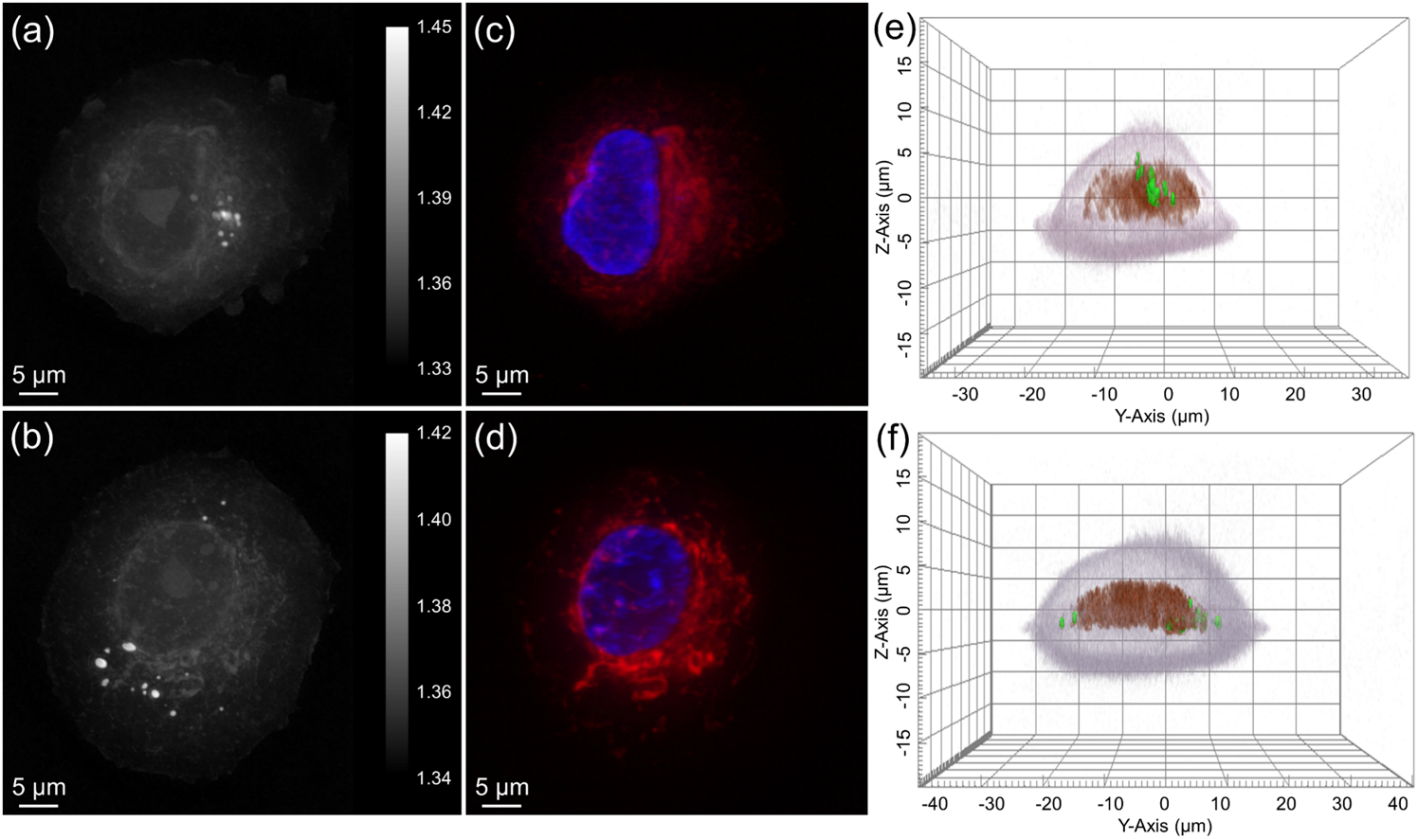
ODT images of MCF-7 cells display the RI distribution of cellular components, with the color scale bar indicating RI values: (a) without and (b) with ultrasound. Fluorescence images of the MCF-7 cell highlighting the nucleus (blue) and mitochondria (red): (c) without and (d) with ultrasound. 3D reconstruction of MCF-7 cell based on RI data, illustrating the cytoplasm (gray), nuclear membrane (red), and lipid droplets (green): (e) without and (f) with ultrasound.

Fig. 8 shows ODT images comparing MCF-7 cells exposed to ultrasound and control cells without irradiation. The RI distribution of cellular components enabled clear visualization of the cell membrane (Fig. 8a and b). The RI values of different cellular components were characterized, showing that the cell membrane exhibited an RI range of 1.33–1.34, the nucleus ranged from 1.35–1.36, and lipid droplets had an RI exceeding 1.37. Cell validation was performed using DAPI and MitoTracker staining (Fig. 8c and d), followed by three-dimensional (3D) computational analysis to assess cellular volume and surface area (Fig. 8e and f).

**Fig. 9:**
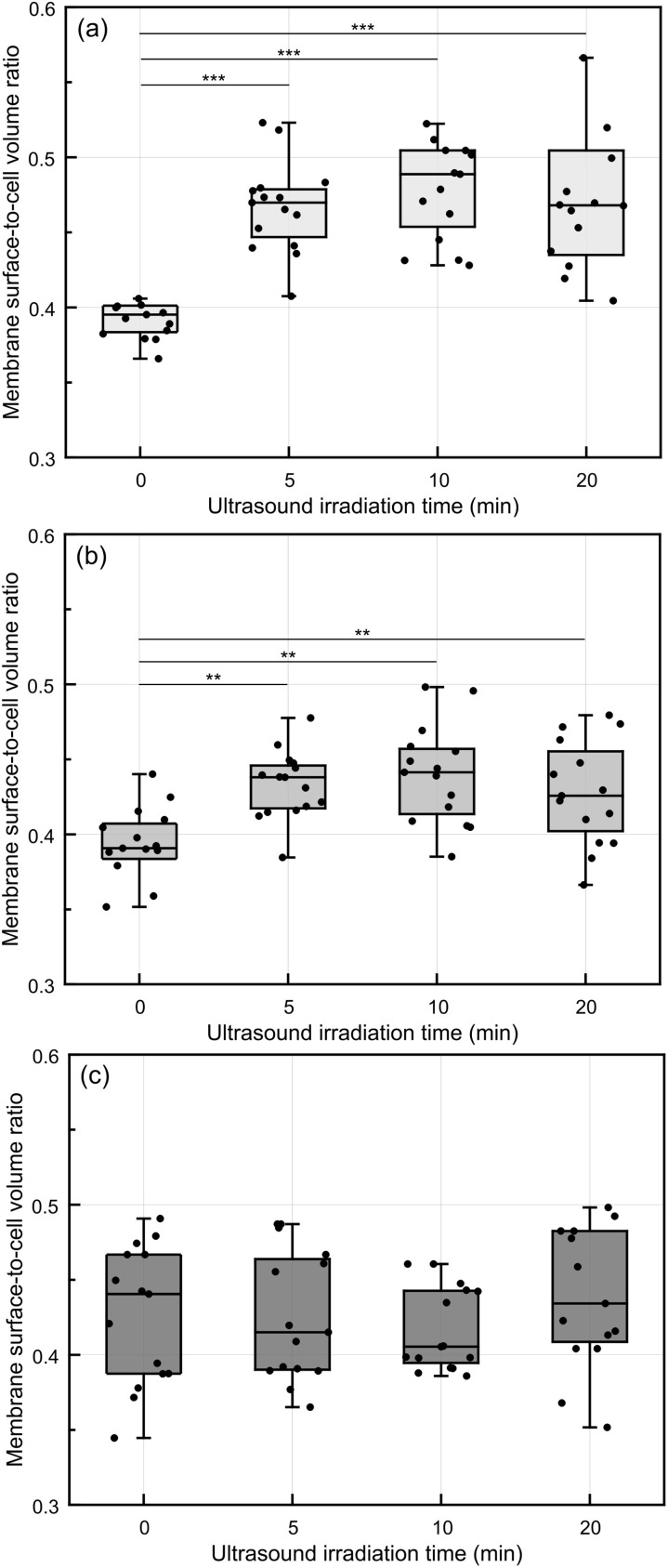
Membrane surface-to-cell volume ratio at different post-ultrasound irradiation time points. (a) Immediately after ultrasound irradiation, (b) 3 h after ultrasound irradiation and (c) 24 h after ultrasound irradiation (*: *p* < 0.05, **: *p* < 0.01, ***: *p* < 0.001, *n* = 3).

The area-to-volume ratio was higher in the ultrasound-irradiated group compared to the control group without ultrasound irradiation. Specifically, immediately post-ultrasound irradiation, the area-to-volume ratio exhibited an increase of over 16.8%, decreasing to an 11% increase at 3 h. By 24 h post-treatment, both the irradiated and non-irradiated groups showed similar area-to-volume ratios (Fig. 9). For accuracy, a minimum of 15 individual cells per group were analyzed by ODT, and the average values were calculated to ensure robustness in detecting single-cell responses.

In addition, ultrasound irradiation led to increases in the cell area-to-volume ratio, suggesting membrane alterations associated with sonoporation. The influence of ultrasound irradiation on cellular morphology and uptake diminished over time, with all groups showing similar area-to-volume ratios by 24 h post-ultrasound irradiation (Fig. 9c). These observations were consistent with previous studies, which reported that sonoporation-induced pore formation typically resolves within min post-ultrasound irradiation.^62,63^ Since the number of ultrasound-induced pores persisting at 24 h is notably reduced compared to immediate post-ultrasound irradiation (Fig. 9c), our findings showed only a marginal increase in nanoparticle internalization in the ultrasound-irradiated groups after 24 h.

## Experimental

### Cell culture and GNP preparation for cells

The human breast adenocarcinoma cell line MCF-7 was obtained from the Korean Cell Line Bank and cultured in RPMI-1640 medium supplemented with 10% fetal bovine serum and 1% penicillin–streptomycin. MCF-7 cells were maintained at 37 °C in a humidified incubator with 5% CO_2_ and seeded in 6-well plates at a density of 1 × 10^6^ cells per well for ICP-AES analysis. GNPs, free from reactants and stabilized in 0.1 mM phosphate-buffered saline (PBS), were obtained from Sigma-Aldrich (USA). GNPs were added to the cell suspension at a final concentration of 20 µg mL^−1^. The MCF-7 cells were incubated with GNPs for 3 h at 37 °C in an incubator with 5% CO_2_ before ultrasound irradiation.

### Nanoparticle tracking analysis for GNP characterization

NTA was performed using a Nanosight Pro instrument (Malvern Panalytical, UK) in light scatter mode (642 nm). Calibration beads and nanoparticle samples were diluted in PBS and distilled water (DW), respectively, to a final volume of 1 mL. Calibration was conducted for the measurement mode. For scatter-mode calibration, 100 nm polystyrene beads were diluted at a ratio of 1 : 499 (v/v). For GNP size analysis, the GNP solution was diluted to a concentration of 1 × 10^9^ particles per mL using DW. The capture settings were as follows: flow rate 3.0 µL min^−1^, exposure time 0.6 ms, number of captures 5, and single capture duration 11.5 s (750 frames). All measurements were carried out at a constant temperature of 21.2 °C. Data analysis was conducted using NS Xplorer software. The diameter distribution was mathematically fitted using a log-normal distribution function, and the fitting procedure was carried out using MATLAB R2025a.

### CCK-8 assay for cytotoxicity evaluation

The cytotoxicity of the GNPs towards MCF-7 cells was assessed using a CCK-8 assay. MCF-7 cells were seeded into 96-well plates and allowed to adhere for 24 h. GNPs were then added at concentrations of 2.5, 5, 10, and 20 µg mL^−1^. Following 24 h of incubation with GNPs, the cell viability was measured by adding an equal volume of CCK-8 solution to each well. The plates were further incubated for 1 h at 37 °C, and the absorbance was measured using a microplate reader (Synergy H1, BioTek Instruments, USA). To control for nanoparticle interference, wells containing medium and GNPs at each tested concentration but no cells were prepared and processed identically; absorbance measured from these particle-only wells was subtracted from sample readings prior to normalization. All experiments were conducted in three independent biological replicates.

### Ultrasound irradiation system

**Fig. 10:**
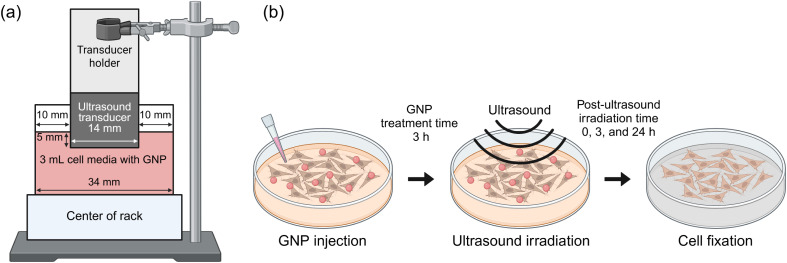
Ultrasound experimental setup: (a) ultrasound transducer scheme. The ultrasound transducer was immobilized at the center of the support rack and fixed with a clamp. The transducer was submerged in the medium to a depth of 5 mm. (b) Timeline illustrating the sequence of GNP treatment, ultrasound irradiation, and subsequent cellular fixation. Following cell culture, GNPs were added to the cell medium and incubated for 3 h before ultrasound irradiation. Ultrasound irradiation was applied for 0, 5, 10, and 20 min for each cell group. The time intervals between ultrasound irradiation and cell fixation were set at 0, 3, and 24 h. Total GNP treatment time was 3, 6, and 27 h for irradiated cells. Created with http://BioRender.com.

Ultrasound irradiation was performed using a transducer (V40AW14B, Vurch Co., South Korea) operating at a frequency of 40 kHz (±2%) with a 14 mm diameter. The ultrasound transducer was submerged into the cell culture medium to a 5 mm depth and used to irradiate the samples (Fig. 10a). The temperature of the culture medium remained stable throughout the irradiation (±1 °C). GNP-treated cells were exposed to ultrasound for durations of 5, 10, and 20 min. Post-ultrasound irradiation, the cells were fixed with 4% paraformaldehyde (PFA) at three different time points: 0 (immediately), 3, and 24 h after irradiation to assess the time-dependent effects of ultrasound on intracellular GNPs (Fig. 10b).

### Label-free GNP detection with confocal microscopy

The MCF-7 cells were seeded in confocal dishes at a density of 1 × 10^5^ cells per dish. The experimental groups for confocal image analysis consisted of a non-irradiated control group and ultrasound-irradiated groups exposed for 5, 10, and 20 min. For confocal imaging, the cells were stained with 4′,6-diamidino-2-phenylindole (DAPI) for nuclear visualization and MitoTracker for mitochondrial labeling. Confocal imaging was performed using a Nikon A1+ inverted confocal microscope (AR HD25, Nikon Corporation, Japan). To configure the reflectance imaging mode in NIS-Elements software, the primary dichroic mirror was set to B520/80 to allow appropriate transmission. Reflectance signals were acquired on channel 4 using the 488 nm laser, with all channel light paths set to “through” mode. To enhance image resolution, the pinhole diameter was reduced to 0.3 Airy units. The final lateral resolution of the acquired images was determined in the confocal software (NIS-Elements software) to be approximately 200 nm. Images were captured at 1024 × 1024 pixels. DAPI fluorescence was excited at 405 nm, and MitoTracker signals were collected following excitation with a 561 nm laser. All imaging settings, including laser power and gain, were maintained constant across samples to ensure reproducibility. Nanoparticle segmentation was performed on normalized image sets from samples. *K*-Means clustering combined with intensity thresholding was applied to accurately identify intracellular GNP regions which was made as previously developed.^64^ Intracellular GNP distribution was evaluated by examining a total of 50 microscopic images (ten representative images per group). All image processing and size measurement were conducted using MATLAB R2024a. The resulting particle size distribution histogram was analyzed by fitting the data to a log-normal distribution function. The result fitting was performed using the Levenberg–Marquardt algorithm to precisely determine the mean cluster size and the standard deviation.

### GNP quantification *via* ICP-AES

ICP-AES was employed for the quantification of intracellular gold elements, recognized as the gold standard technique for nanoparticle measurement. The ICP-AES instrument used (OPTIMA 8300, PerkinElmer, USA) operated with an argon plasma source at 6000 K. The system offered a spectral range of 167–782 nm with a resolution of 0.006 nm at 200 nm, and a detection limit of 10 ppb, suitable for detecting 50 nm GNPs. For the analysis, 2 mL of the initial GNP solution was diluted to 10 mL, resulting in a dilution factor of 5, which was applied to adjust the ICP-AES results. The average intracellular gold element concentrations were divided by the total cell count. The relationship between the measured gold element and the number of nanoparticles is described using the following equation:^65^1
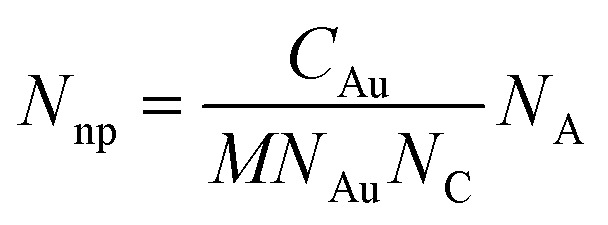
where *N*_np_ is the number of GNPs, *C*_Au_ is the gold element concentration (196.966657 g mol^−1^), *M* is the molar mass of GNPs, *N*_Au_ represents the number of atoms in a 50 nm GNP (3 849 991), *N*_C_ is the total number of cells (1 × 10^6^ cells), and *N*_A_ is Avogadro's number (6.022 × 10^23^ mol^−1^). This equation was utilized to calculate the intracellular GNP quantity, providing an accurate quantification of nanoparticle uptake.

The nanoparticle uptake data obtained by ICP-AES were modeled using a first-order absorption model, which effectively allowed more accurate representation of the uptake kinetics.^66^

2 *Y*(*t*) = *A*_max_(1 − e^−*kt*^)

where *A*_max_ is the maximum absorption (calculated value from each group), *k* is the absorption constant, and *t* is the time. This equation was used for the first-order absorption model fitting for ICP-AES results.

### Confirmation of sonoporation *via* ODT

For ODT analysis, the MCF-7 cells were seeded in a TomoDish at a density of 5 × 10^4^ cells. Image analysis was performed on four groups: non-irradiated control and groups exposed to ultrasound for 5, 10, and 20 min. ODT is a three-dimensional imaging technique that measures the refractive index (RI) tomogram of cells using laser-based quantitative phase imaging. This approach quantifies phase shifts as a laser passes through transparent cell structures. ODT images of single MCF-7 cells were acquired using an HT-1H holotomographic microscope (Tomocube Inc., South Korea), DAPI, and MitoTracker staining was used for appropriate localization of the cells. For the surface area-to-volume ratio, 15 different cell images for every experimental group (0, 5, 10, and 20 min ultrasound irradiation and 0, 3, and 24 h post-ultrasound irradiation) were acquired. The images were then processed in the TomoAnalysis™ software for quantitative analysis of cellular and membrane properties. Sonoporation levels in the cellular membrane were assessed by the membrane surface area-to-volume ratio.

### Statistical analysis

Statistical significance was determined using one-way ANOVA followed by Tukey's HSD *post hoc* test. Data are represented as the mean ± SD. A *p*-value of less than 0.05 (*p* < 0.05) was considered statistically significant.

## Conclusions

This study confirmed the spatiotemporal dynamics of GNPs in MCF-7 cells through label-free tracking and quantitative analysis. Ultrasound irradiation promoted the dispersion of intracellular GNPs by reducing their aggregation size by a few tens of percent in our experimental setup. It significantly enhanced internalization with the disaggregation effect being more prominent at low-frequency ultrasound than MHz ultrasound. Additionally, sonoporation was observed following ultrasound irradiation, with its effects reducing over time as the post-ultrasound irradiation period progressed. The influence of ultrasound on cellular uptake was diminished notably after 3 h, indicating a rapid but transient effect. Previous studies reported that the release rate of molecules from GNP-conjugated liposomes, gold nanoshell complexes, and mesoporous silica-coated gold nanorods was relatively low.^67–69^ On the contrary, our findings suggest that ultrasound irradiation may facilitate rapid drug delivery within a narrow time window following administration, potentially enhancing the efficiency of drug or nanoparticle distribution. Although GNPs are primarily trafficked to the perinuclear region due to endosomal and lysosomal transport, ultrasound exposure can reduce the size of nanoparticle clusters, potentially leading to therapeutic advantages.

## Author contributions

Jiwon Kim: conceptualization, methodology, investigation, visualization, writing – original draft, writing – review & editing. Chaewon Bae: methodology, investigation, data curation, methodology, writing – review & editing. Rodrigo Hernández Millares: methodology, investigation, writing – review & editing. Taeyun Kim: investigation, writing – reviewing. Yejin Lee: investigation, writing – reviewing. Kangwon Lee: validation, writing – reviewing. Sung-Joon Ye: supervision, conceptualization, validation, writing – reviewing and editing, funding acquisition, project administration.

## Conflicts of interest

The authors confirm that the research was carried out without any commercial or financial interest.

## Data Availability

All relevant data are provided within the manuscript and its additional files. Additional data are available upon reasonable request.
